# Competitive Risk Model for Specific Mortality Prediction in Patients with Bladder Cancer: A Population-Based Cohort Study with Machine Learning

**DOI:** 10.1155/2022/9577904

**Published:** 2022-08-25

**Authors:** Hao Su, Xiaoqiang Xue, Yutao Wang, Yi Lu, Chengquan Ma, Zhigang Ji, Xiaozhe Su

**Affiliations:** ^1^Department of Urology, Peking Union Medical College Hospital, Peking Union Medical College, Chinese Academy of Medical Sciences, Beijing 100730, China; ^2^Department of Urology, Renmin Hospital of Wuhan University, Wuhan 430060, China

## Abstract

**Background:**

Noncancer death accounts for a high proportion of all patients with bladder cancer, while these patients are often excluded from the survival analysis, which increases the selection bias of the study subjects in the prediction model.

**Methods:**

Clinicopathological information of bladder cancer patients was retrieved from the Surveillance, Epidemiology, and End Results (SEER) database, and the patients were categorized at random into the training and validation cohorts. The random forest method was used to calculate the importance of clinical variables in the training cohort. Multivariate and univariate analyses were undertaken to assess the risk indicators, and the prediction nomogram based on the competitive risk model was constructed. The model's performance was evaluated utilizing the calibration curve, consistency index (C index), and the area under the receiver operator characteristic curve (AUC).

**Results:**

In total, we enrolled 39285 bladder cancer patients in the study (27500 patients were allotted to the training cohort, whereas 11785 were allotted to the validation cohort). A competitive risk model was constructed to predict bladder cancer-specific mortality. The overall C index of patients in the training cohort was 0.876, and the AUC values were 0.891, 0.871, and 0.853, correspondingly, for 1-, 3-, and 5-year cancer-specific mortality. On the other hand, the overall C index of patients in the validation cohort was 0.877, and the AUC values were 0.894, 0.870, and 0.847 for 1-, 3-, and 5-year correspondingly, suggesting a remarkable predictive performance of the model.

**Conclusions:**

The competitive risk model proved to be of great accuracy and reliability and could help clinical decision-makers improve their management and approaches for managing bladder cancer patients.

## 1. Introduction

Bladder cancer is a malignancy that occurs in the bladder mucosa. In terms of incidence rate, it is only slightly lower than prostate cancer, making it the second most prevalent malignancy invading the urinary system. The Global Cancer Observatory (GLOBOCAN), a report produced by the World Health Organization, estimates that bladder cancer represents about 3% of all cancer diagnoses globally, with the highest proportion occurring in industrialized countries. In male, bladder cancer has been identified to be the sixth most prevalent malignancy [1]. The American Cancer Society reported that in 2022, there were approximately 81180 newly diagnosed cases of bladder cancer in the United States (U.S.), of which 61700 were male, ranking fourth in new cases of male cancer, and the estimated number of bladder cancer-caused deaths was about 17100, of which 12120 were male [2]. Most of the bladder cancer cases are urothelial carcinomas in pathological classification, and a few are squamous cell carcinomas and other pathological types [3]. Bladder cancer can be categorized into two subtypes: myometrial invasive bladder cancer (MIBC) and non-muscle-invasive bladder cancer (NMIBC), with the latter attributed to roughly 75% of the patients. Half of the NMIBCs are of low pathological differentiation, whereas most of MIBCs are of high atypia [4, 5]. Smoking and occupational exposure to carcinogens (such as aromatic amines, polycyclic aromatic hydrocarbons, and chlorinated hydrocarbons) are important risk factors for bladder cancer [6]. Bladder cancer can pose a heavy social and economic burden on patients and remains a great challenge for global public health [7].

Identification of risk factors for cancer death and noncancer death is crucial for individualized cancer treatment. Noncancer death is also an important cause of death in cancer patients [8]. Studies have found that multiple noncancer factors, including those concomitant with other cancers, circulatory diseases, nondisease causes, other noncancer diseases, and respiratory diseases, also largely contributed to the deaths of bladder cancer patients, and the proportion of noncancer causes is increasing. Therefore, management of other complications is critical when treating bladder cancer patients [9]. Conventional prediction analyses for the prognosis, recurrence, survival, and mortality of bladder cancer depend upon the number of cancer sites, cancer size, recurrence rate, pathological types, in situ cancer, and TNM stages, which cannot provide predictions that are individualized, precise, and applicable for the patients [10, 11]. As a statistical model-based prediction method, the nomogram has some remarkable merits compared with other approaches and could produce a more scientific prognostic prediction for bladder cancer patients [12, 13].

The goal of this research was to build a competitive risk model for the prediction of noncancer deaths among bladder cancer patients and evaluate its predictive accuracy based on the SEER database, so as to provide a reference for clinical decisions.

## 2. Methods

### 2.1. Data Source

We collected clinicopathological data from patients who registered in the SEER program of the National Cancer Institute. SEER is a publicly accessible database that collects clinical, demographic, and outcome data of patients with all types of malignancies, with 18 registries covering 30% of the U.S. population. All the data used in this study followed the specifications of the SEER database. No interventions or patients' privacies were involved in this study, so ethical approval and informed consent were not needed.

Patients with bladder cancer who registered from 2010 to 2015 were identified in accordance with the third version of the International Classification of Diseases for Oncology (ICD-O-3). Collected clinicopathological information mainly included age (age at diagnosis), marriage (marital status at diagnosis), sex (male or female), race (race recode (white, black, other)), primary (primary site-labeled), grade (grade), behave (ICD-O-3 hist/behave), stage (derived AJCC stage group 7th ed (2010–2015)), T (derived AJCC T 7th ed (2010–2015)), N (derived AJCC N 7th ed (2010–2015)), M (derived AJCC M 7th ed (2010–2015)), surgery (RX Summ-surg prim site (1998+)), chemotherapy (chemotherapy recode (yes, no/unk)), radiation (radiation sequence with surgery), positive (regional nodes positive (1988+)), bone (SEER combined mets at DX-bone (2010+)), brain (SEER combined mets at DX-brain (2010+)), liver (SEER combined mets at DX-liver (2010+)), lung (SEER combined mets at DX-lung (2010+)), size (CS tumor size (2004–2015)), number (total number of in situ/malignant tumors for patient), time (survival months), and status (COD to site rec KM).

### 2.2. Data Exclusion Criteria

Although SEER includes a large number of cancer records, but there are still many missing values in the registration process. Interpolation could be difficult when there is a large proportion of missing values. Therefore, patients meeting the following criteria were excluded:

1. Age at diagnosis less than 18 years old
2. Marital status at diagnosis unavailable
3. Race unknown
4. Derived AJCC stage group coded as NA or UNK stage
5. Cancer stages unknown
6. Regional nodes positive (1988+) coded as 99
7. CS tumor size (2004–2015) coded as 999
8. Survival months was 0
9. SEER combined mets at DX-bone (2010+) coded as unknown or N/A
10. SEER combined mets at DX-brain (2010+) coded as unknown or N/A
11. SEER combined mets at DX-liver (2010+) coded as unknown or N/A
12. SEER combined mets at DX-lung (2010+) coded as unknown or N/A

After screening the collected data, 39285 patients were recruited for this analysis.

### 2.3. Data Analysis

Patients who died of bladder cancer were set as events of interest, those who died of other causes were set as competitive events, and survival or loss to follow-up as deletion events.

Before modeling, nonrepetitive random sampling was conducted according to the common 7 : 3 ratio in the risk model to generate the training as well as the validation cohorts. First, the random forest method was used to calculate the importance of clinical variables in the training cohort, the variables with high importance were used in the subsequent study of the competitive risk model. Subsequently, the univariate competitive risk model was used in the training cohort, and multivariable analysis was carried out on variables with *P* values less than 0.1 [14]. Then, variables with *p* < 0.1 in multivariate analysis were selected to construct the predictive nomogram of bladder cancer-specific mortality. A competitive risk model for bladder cancer-specific mortality was constructed, and a predictive nomogram was plotted.

To examine the prediction performance (accuracy) of the model, we utilized the c-statistic and calibration curve. The R 4.0.4 software (R Development Core Team, Vienna, http://www.R-project.org) was utilized to execute all analyses of statistical data. The crr function in the algorithm integration package ‘cmprsk' was utilized to complete the competitive risk model, whereas the cuminc tool was utilized to conduct the fine-gray test [15].

## 3. Results

### 3.1. Clinical Characteristics of Included Patients

There were 108884 patients identified who received bladder cancer diagnoses between 2010 and 2015. After screening by eligibility requirements, 39285 patients were incorporated, with the longest follow-up period of 83 months and the median period of follow-up of 29 months. There were 13086 patients who died, among whom 6571 died of bladder cancer and 6515 of other causes. Patients were allotted at random to either the training cohort (*n* = 27500) or the validation cohort (*n* = 11785). The patients had a mean age of 70.82 ± 11.84 years old. Among them, 24669 (62.79%) were married, 29573 (75.28%) males, and 35133(89.43%) were white. There were 37443 patients (95.31%) who had no regional lymph node biopsy or negative biopsy results. As for the primary cancer site, there were 12142 (30.91%) on bladder NOS and 9833 (25.03%) on the lateral wall of the bladder. There were 18240 (46.43%) patients with grade IV cancer and 9797 (24.94%) with grade II. The number of papillary transitional cell carcinomas was 26737 (68.06%). For cancer stages, 16978 patients (43.22%) were in stage 0a or Ois, and 9963 patients (25.36%) were in stage I. For surgery of the primary site, 25288 patients (64.37%) underwent excisional biopsy. For cancer size, 15060 patients (38.34%) had tumor sizes of less than 3 cm. As for the number of tumors, 25415 patients (64.69%) were diagnosed with a single tumor.  Table 1 presents the detailed clinical features of the patients who were included in the research.

### 3.2. Clinical Variables Determination by Random Forest in Train Cohort

**Figure 1:**
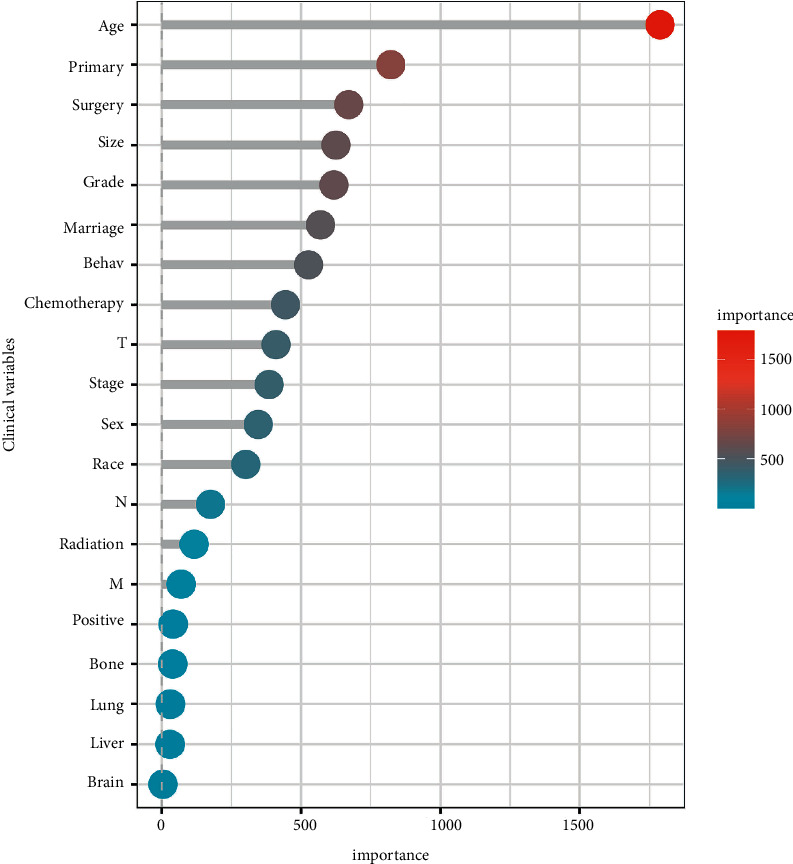
Important parameters of the clinical variables by random forest.

The RandomForest packages were applied to evaluate the importance of the clinical variables, and the random seed was set as 123. Age, primary, surgery, size, and grade were considered to be characteristic representative variables in the training cohort, and their importance parameters of random forest screening are shown in Figure 1.

### 3.3. Construction of the Competitive Risk Model

**Figure 2:**
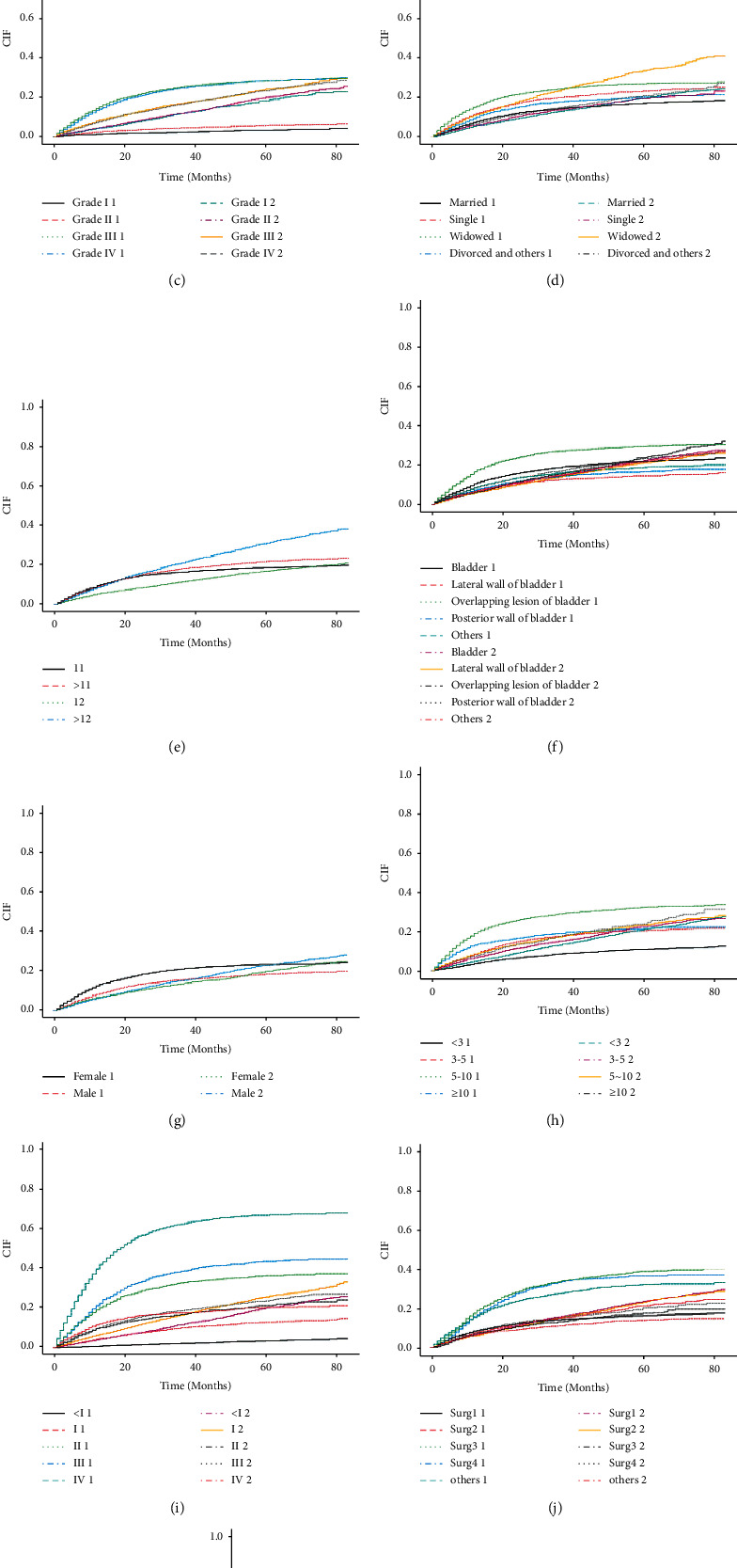
Cumulative mortality at different levels of independent influencing factors included in the model. (a to k denote behaviour, chemotherapy, grade, marriage, number, primary, sex, size, stage, surgery, and T, respectively).

**Figure 3:**
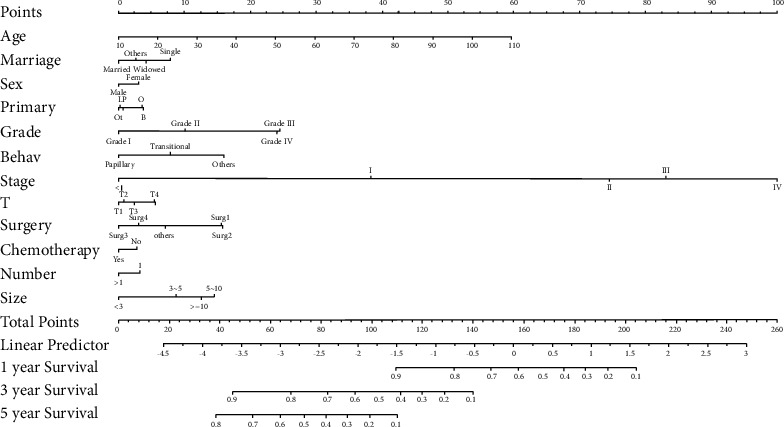
Nomogram for predicting tumor-specific mortality in bladder cancer. (B, L, O, P, and Ot in primary denote bladder, NOS, lateral wall of bladder, overlapping lesion of bladder, posterior wall of bladder, and others, respectively). Papillary and transitional in behaviour denote papillary transitional cell carcinoma and transitional cell carcinoma, NOS, respectively).

The five clinical variables that affect the importance close to 0 in the random forest were excluded, and the univariate and multivariate analyses were performed on the remaining clinical variables. We constructed a univariate competitive risk model in the training cohort, and the results showed that all variables were statistically significant (*p* < 0.1). All parameters in this univariate model were incorporated into the multivariate competitive risk model, and the results showed that age at diagnosis, marital status at diagnosis, sex, primary site-labeled, grade, ICD-O-3 hist/behav, derived AJCC stage group 7th ed (2010–2015), derived AJCC T 7th ed (2010–2015), RX Summ-surg prim site (1998+), chemotherapy recode (yes, no/unk), CS tumor size (2004–2015), and total number of in situ/malignant tumors for patients were independent risk indicators for bladder cancer-specific mortality. Therefore, we incorporated the above variables to construct a competitive risk model for predicting tumor-specific mortality (Table 2; Figures 2 and 3).

### 3.4. Model Validation

**Figure 4:**
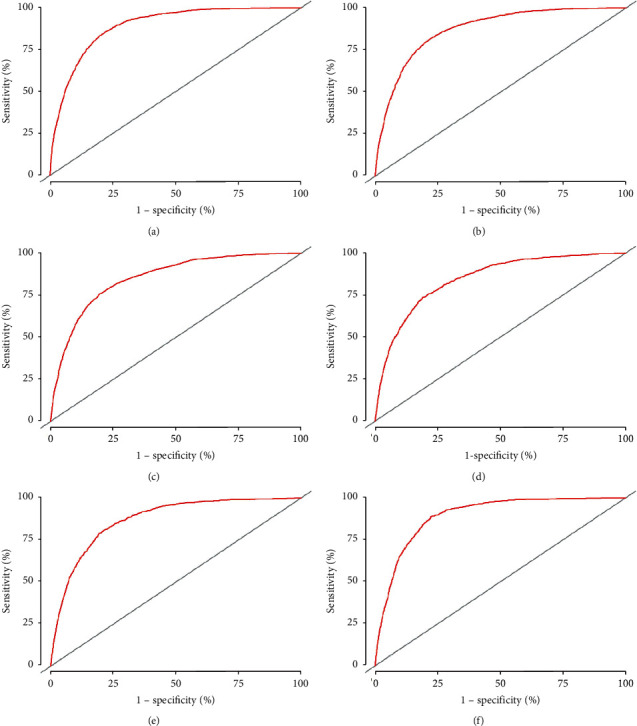
ROC curves of 1-, 3-, and 5-year cancer-specific mortality in the training cohort and validation cohort. (a, b, and c are ROC curves of 1-, 3-, and 5-year cancer-specific mortality in the training cohort, respectively; d, e, and f are ROC curves of 1-, 3-, and 5-year cancer-specific mortality in the validation cohort, respectively).

**Figure 5:**
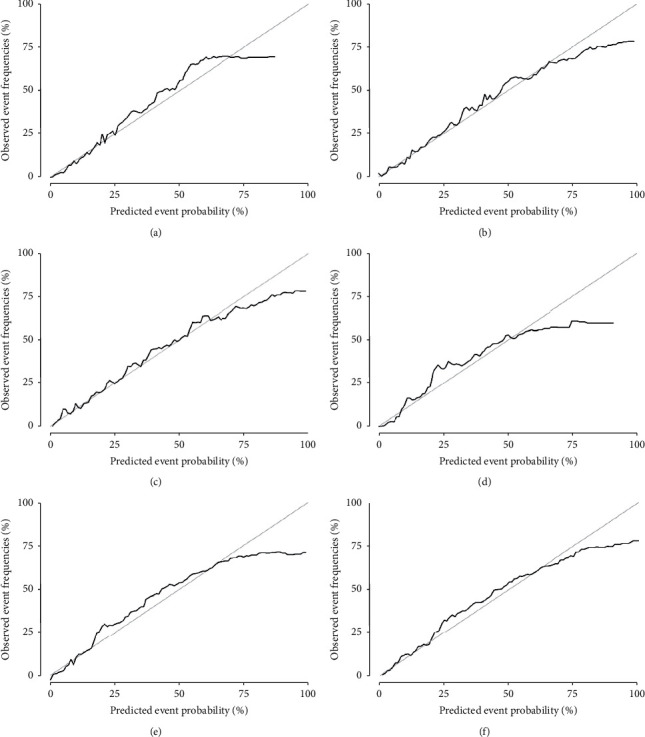
Calibration curves of 1-, 3-, and 5-year cancer-specific mortality in the validation cohort and validation cohort. (a, b, and c are calibration curves of 1-, 3-, and 5-year cancer-specific mortality in the training cohort, respectively; d, e, and f are calibration curves of 1-, 3-, and 5-year cancer-specific mortality in the validation cohort, respectively).

The C index was adopted to assess the accuracy of training and validation cohorts in the model. In the training cohort, the model predicted that the overall C index of patients was 0.876, and the areas under the ROC curve (AUC) were 0.891, 0.871, and 0.853 correspondingly for 1-, 3-, and 5-year cancer-specific mortality. In the validation cohort, the overall C index of patients was 0.877, and the AUC values were 0.894, 0.870, and 0.847 correspondingly for 1-, 3-, and 5-year cancer-specific mortality, which indicated that the model was of great prediction performance. The details are shown in Figure 4 and Figure 5. In addition, the calibration curve of the model illustrated that the anticipated value of the model was almost identical to the actual observation value, illustrating the considerable accuracy of the model.

## 4. Discussion

Bladder cancer is a prevalent malignancy in the urinary system with its incidence rate only second to that of prostate cancer, presenting a great threat to public health. Currently, individualized cancer treatment has been taken more seriously, and accurate prediction of the survival, prognosis, and mortality of bladder cancer patients is of great importance. Conventional survival analyses (e.g., the Cox proportional hazards model and the Kaplan–Meier marginal regression) usually include only one endpoint event, such as death. However, when there are multiple events and these events compete with each other, the use of a single endpoint analysis would lead to deviations in the anticipated probability of endpoint events. In this research, we employed a competitive risk model to evaluate the risk variables that affect bladder cancer patients' prognoses. Although this model accounts for bladder cancer-related mortality, it also takes into consideration the deaths attributed to other forms of cancer as well as other events.

Nomograms for prognosis prediction of bladder cancer are drawing increasing attention recently. There have been plenty of studies that applied nomograms to anticipate the overall survival (OS) of bladder cancer patients. Zhan et al. plotted a nomogram that was highly differentiated and accurate and constructed a relevant risk categorization system to anticipate the cancer-specific survival probability for MIBC patients who underwent partial cystectomy [16]. Zhan et al. also constructed a nomogram to provide an accurate prognostic prediction for cancer-specific survival probability in patients with lymph node-positive bladder cancer [17]. Tao et al. created a prognostic nomogram to anticipate the OS in patients with distant-metastatic bladder cancer (DMBC) [18]. Wang et al. applied a nomogram to predict the influence of previous cancer on the OS rate of bladder cancer patients [19]. Yang et al. plotted a prognostic nomogram to anticipate cancer-specific survival in patients with bladder urothelial carcinoma following radical cystectomy [20]. Many other studies have also constructed nomograms of overall survival in patients with bladder cancer [21, 22].

A nomogram to anticipate individual cancer-specific mortality was established in this research premised on a sizable cohort of bladder cancer patients from the SEER database. The nomogram was constructed based on demographic, pathological, and surgical data, which showed remarkable effects in both the training and the validation cohorts, illustrating that the nomogram is of clinical applicability for predicting bladder cancer-specific mortality. Our model contained the following variables produced from clinical practice: age at diagnosis, marital status at diagnosis, sex, primary site-labeled, grade, ICD-O-3 hist/behav, derived AJCC stage group 7th ed (2010–2015), derived AJCC T 7th ed (2010–2015), RX Summ-surg prim site (1998+), chemotherapy recode (yes, no/unk), CS tumor size (2004–2015), the total number of in situ/malignant tumors for patients, in which age was a significant risk variable for the bladder cancer-specific mortality, suggesting that the risk of death in bladder cancer patients would increase significantly with age. Prognostic analyses for bladder cancer conducted by other studies also showed that age played a crucial role in cancer death, and the death rate in patients increased with the increase of age at diagnosis [23, 24]. Another risk factor appeared to be sex. Female bladder cancer patients experienced a poorer prognosis in contrast with males, which was consistent with the results of other studies that found female patients with bladder urothelial carcinoma had higher cancer-specific mortality [25, 26]. The cancer stage was also proved to be a substantial risk indicator for the prognosis of bladder cancer. Upgrading of the stage was associated with a worse prognosis, which was also consistent with many other studies. Cancer T staging was an important part of the model. Many research reports illustrated that bladder invasion depth was strongly linked to bladder cancer patients' prognoses. With the progress of T staging, cancer would be more invasive and progressive [27]. On the other hand, the prognosis of bladder cancer patients with distant organ metastasis was highly unfavorable in contrast with those without metastasis. Studies showed that different distant metastasis sites had different effects on mortality, and the mortality of patients with multiple distant metastasis sites was significantly higher than those with single distant metastasis sites [28, 29]. Furthermore, the nomogram also illustrated that an increase in cancer size resulted in a poorer prognosis in bladder cancer patients. Other studies have further confirmed the influence of cancer size on bladder cancer-specific prognosis [30]. Moreover, the number of cancers is also related to the prognosis of bladder cancer. Patients with multiple bladder cancer had a poorer prognosis than those with single bladder cancer.

In most cases, conventional survival analysis techniques only take into account the findings of one endpoint and thus might overstate or undervalue the impact of independent risk variables, which are often present in right-censored data and are commonly employed in survival analysis methods. The competitive risk model is applicable to the survival data of multiple endpoints. It is an analytical method to deal with the survival data of multiple potential outcomes. If the clinical survival data have multiple outcomes and the hypothesis of “deletion independence” is not satisfied when there are competitive outcomes, the Cox proportional risk model cannot be used for multifactor analysis, otherwise the wrong hazard ratio will occur. At this time, the competitive risk model reflects its unique value. However, this study has some limitations. First, the prognosis of individuals with bladder cancer is likely influenced by their lifestyle, genes, and other factors; however, the SEER database did not include information on these variables. Second, only internal validation was performed for our model. Further validation of external clinical data and future clinical applications are needed. It is expected that future studies will be conducted in order to investigate other aspects.

## 5. Conclusion

We constructed a competitive risk model to anticipate cancer-specific mortality in bladder cancer patients, which proved to be of great accuracy and reliability and could help clinical decision-makers improve the management and follow-up methods for these patients.

## Figures and Tables

**Table 1:** Clinical features of the patients.

Factors	Training cohort (*N* = 27500)	Validation cohort (*N* = 11785)	All data (*N* = 39285)
*Age*	70.81 ± 11.84	70.85 ± 11.83	70.82 ± 11.84

*Marriage*			
Married	17338 (63.05)	7331 (62.21)	24669 (62.79)
Single	3257 (11.84)	1395 (11.84)	4652 (11.84)
Widowed	4056 (14.75)	1752 (14.87)	5808 (14.78)
Divorced and others	2849 (10.36)	1307 (11.09)	4156 (10.58)

*Sex*			
Female	6780 (24.65)	2932 (24.88)	9712 (24.72)
Male	20720 (75.35)	8853 (75.12)	29573 (75.28)

*Race*			
White	24597 (89.44)	10536 (89.4)	35133 (89.43)
Black	1597 (5.81)	692 (5.87)	2289 (5.83)
Other	1306 (4.75)	557 (4.73)	1863 (4.74)

*Positive*			
No nodes/Negative	26222 (95.35)	11221 (95.21)	37443 (95.31)
Positive	1278 (4.65)	564 (4.79)	1842 (4.69)

*Primary*			
*Bladder, NOS*	8519 (30.98)	3623 (30.74)	12142 (30.91)
Lateral wall of bladder	6831 (24.84)	3002 (25.47)	9833 (25.03)
Overlapping lesion of bladder	3416 (12.42)	1408 (11.95)	4824 (12.28)
Posterior wall of bladder	2990 (10.87)	1271 (10.78)	4261 (10.85)
Others	5744 (20.89)	2481 (21.05)	8225 (20.94)

*Grade*			
Grade I	3281 (11.93)	1444 (12.25)	4725 (12.03)
Grade II	6854 (24.92)	2943 (24.97)	9797 (24.94)
Grade III	4555 (16.56)	1968 (16.7)	6523 (16.6)
Grade IV	12810 (46.58)	5430 (46.08)	1824 0(46.43)

*Behaviour*			
Papillary transitional cell carcinoma	18727 (68.1)	8010 (67.97)	26737 (68.06)
Transitional cell carcinoma	7284 (26.49)	3139 (26.64)	10423 (26.53)
Others	1489 (5.41)	636 (5.4)	2125 (5.41)

*Stage*			
<I	11834 (43.03)	5144 (43.65)	16978 (43.22)
I	6983 (25.39)	2980 (25.29)	9963 (25.36)
II	4286 (15.59)	1869 (15.86)	6155 (15.67)
III	1903 (6.92)	738 (6.26)	2641 (6.72)
IV	2494 (9.07)	1054 (8.94)	3548 (9.03)

*T*			
T0 and others	11889 (43.23)	5163 (43.81)	17052 (43.41)
T1	7168 (26.07)	3058 (25.95)	10226 (26.03)
T2	5059 (18.4)	2205 (18.71)	7264 (18.49)
T3	2145 (7.8)	891 (7.56)	3036 (7.73)
T4	1239 (4.51)	468 (3.97)	1707 (4.35)

*N*			
N0 and others	25670 (93.35)	10978 (93.15)	36648 (93.29)
N1	704 (2.56)	312 (2.65)	1016 (2.59)
N2	901 (3.28)	402 (3.41)	1303 (3.32)
N3	225 (0.82)	93 (0.79)	318 (0.81)

*M*			
M0	26578 (96.65)	11429 (96.98)	38007 (96.75)
M1	922 (3.35)	356 (3.02)	1278 (3.25)

*Surgery*			
Surg1	17746 (64.53)	7542 (64)	25288 (64.37)
Surg2	3846 (13.99)	1709 (14.5)	5555 (14.14)
Surg3	1446 (5.26)	597 (5.07)	2043 (5.2)
Surg4	2035 (7.4)	923 (7.83)	2958 (7.53)
Others	2427 (8.83)	1014 (8.6)	3441 (8.76)

*Radiation*			
Others	1636 (5.95)	659 (5.59)	2295 (5.84)
No radiation	25864 (94.05)	11126 (94.41)	36990 (94.16)

*Chemotherapy*			
Yes	9288 (33.77)	3874 (32.87)	13162 (33.5)
No	18212 (66.23)	7911 (67.13)	26123 (66.5)

*Bone*			
No	27193 (98.88)	11669 (99.02)	38862 (98.92)
Yes	307 (1.12)	116 (0.98)	423 (1.08)

*Brain*			
No	27474 (99.91)	11775 (99.92)	39249 (99.91)
Yes	26 (0.09)	10 (0.08)	36 (0.09)

*Liver*			
No	27327 (99.37)	11713 (99.39)	39040 (99.38)
Yes	173 (0.63)	72 (0.61)	245 (0.62)

*Lung*			
No	27176 (98.82)	11658 (98.92)	38834 (98.85)
Yes	324 (1.18)	127 (1.08)	451 (1.15)

*Size*			
<3	10526 (38.28)	4534 (38.47)	15060 (38.34)
3∼5	8123 (29.54)	3502 (29.72)	11625 (29.59)
5∼10	7122 (25.9)	2975 (25.24)	10097 (25.7)
≥10	1729 (6.29)	774 (6.57)	2503 (6.37)

*Number*			
1	17821 (64.8)	7594 (64.44)	25415 (64.69)
>1	9679 (35.2)	4191 (35.56)	13870 (35.31)

Surgery 1 to 4 denote, respectively: excisional biopsy, electrocautery, radical cystectomy plus ileal conduit, and radical cystectomy (female only); anterior exenteration.

**Table 2:** Univariate and multivariate competitive risk model.

Factors	*Univariate analysis*			*Multivariate analysis*		
	HR (95%CI)	*Z*	*P*	HR (95%CI)	*Z*	*P*
Age	1.02(1.02–1.03)	16.4	<0.001	1.024(1.021–1.027)	15.152	<0.001
Marriage	1.16(1.13–1.19)	12	<0.001	1.062(1.032–1.092)	4.148	<0.001
Sex	0.74(0.69–0.78)	−9.5	<0.001	0.902(0.84–0.969)	−2.815	0.005
Race	1.10(1.04–1.16)	3.24	0.0012			
Primary	0.98(0.96–1.00)	−2.09	0.037	0.977(0.957–0.998)	−2.197	0.028
Grade	1.98(1.92–2.04)	41.9	<0.001	1.223(1.174–1.275)	9.563	<0.001
Behav	2.77(2.67–2.88)	53.4	<0.001	1.41(1.336–1.488)	12.501	<0.001
Stage	2.21(2.17–2.25)	84.2	<0.001	1.937(1.823–2.058)	21.309	<0.001
T	2.15(2.11–2.20)	78.2	<0.001	1.136(1.078–1.198)	4.725	<0.001
*N*	2.23(2.16–2.31)	46.5	<0.001			
M	9.17(8.39–10.00)	49	<0.001			
Surgery	1.27(1.24–1.29)	25.6	<0.001	0.909(0.884–0.935)	−6.688	<0.001
Radiation	0.29(0.26–0.31)	−31.3	<0.001			
Chemotherapy	0.69(0.64–0.72)	−13	<0.001	1.234(1.153–1.32)	6.069	<0.001
Size	1.57(1.53–1.61)	33.1	<0.001	1.214(1.174–1.255)	11.272	<0.001
Number	1.12(1.05–1.18)	3.65	0.00026	0.916(0.858–0.978)	−2.629	0.009

## Data Availability

The data that support the findings of this study are available from the corresponding author upon reasonable request.
